# Prevalence of rhinitis and associated factors in adolescents and adults: a Global Asthma Network study

**DOI:** 10.1590/1984-0462/2023/41/2021400

**Published:** 2023-03-03

**Authors:** Marilyn Urrutia-Pereira, Lucas Pitrez Mocelin, Philippa Ellwood, Luis Garcia-Marcos, Laura Simon, Pietro Rinelli, Herberto José Chong-Neto, Dirceu Solé

**Affiliations:** a Universidade Federal do Pampa, Bagé, RS, Brazil.; b University of Auckland, Auckland, New Zealand.; c IMIB Bio-health research Institute of Murcia, El Palmar, Murcia, Spain.; d Universidade Federal do Paraná, Curitiba, PR, Brazil.; e Universidade Federal de São Paulo, São Paulo, SP, Brazil.

**Keywords:** Prevalence, Rhinitis, Risk factors, Adolescents, Prevalência, Rinite, Fatores de risco, Adolescentes

## Abstract

**Objective:**

To determine the prevalence of allergic rhinitis and associated factors in adolescents and in their parents/guardians.

**Methods:**

A cross-sectional study, applying a standardized and validated written questionnaire. Adolescents (13–14 years old; n=1,058) and their parents/guardians (mean age=42.1 years old; n=896) living in the city of Uruguaiana, southern Brazil, responded to the Global Asthma Network standard questionnaires.

**Results:**

The prevalence of allergic rhinitis in adolescents was 28.0%, allergic rhinoconjunctivitis, 21.3%, and severe forms of allergic rhinitis, 7.8%. In the adults, the prevalence of allergic rhinitis was 31.7%. Some associated factors with allergic rhinitis in adolescents include low physical exercise (OR 2.16; 95%CI 1.15–4.05), having only one older sibling (OR 1.94; 95CI 1.01–3.72) and daily meat consumption (OR 7.43; 95% CI 1.53–36.11). In contrast, consuming sugar (OR 0.34; 95%CI 0.12–0.93) or olive oil (OR 0.33; 95%CI 0.13–0 .81) once or twice a week, and eating vegetables daily (OR 0.39; 95%CI 0.15–0.99) were considered factors negatively associated. In adults, exposure to fungi at home (OR 5.25; 95%CI 1.01–27.22) and consumption of meat once or twice a week (OR 46.45; 95CI 2.12–1020.71) were factors associated with the medical diagnosis of allergic rhinitis, while low education (OR 0.25; 95%CI 0.07–0.92) was found to be a factor negatively associated.

**Conclusions:**

The prevalence of allergic rhinitis in adolescents is high, as well as its medical diagnosis in adults living in Uruguaiana. Environmental factors, especially food habits, were associated with findings in both groups.

## Resumo

**Objetivo:**

Determinar a prevalência de rinite alérgica e fatores associados em adolescentes e em seus pais/responsáveis.

**Métodos:**

Estudo transversal, com aplicação de questionário escrito padronizado e validado. Adolescentes (13–14 anos; n=1.058) e seus pais/responsáveis (média de idade=42,1 anos; n=896) residentes na cidade de Uruguaiana, Sul do Brasil, responderam aos questionários padrão da Global Asthma Network.

**Resultados:**

A prevalência de rinite alérgica em adolescentes foi de 28,0%, a de rinoconjuntivite alérgica foi de 21,3% e a de formas graves de rinite alérgica, de 7,8%. Nos adultos, a prevalência de rinite alérgica foi de 31,7%. Alguns fatores associados à rinite alérgica em adolescentes incluem fazer pouco exercício físico (*odds ratio* — OR 2,16; intervalo de confiança — IC95% 1,15–4,05), ter apenas um irmão mais velho (OR 1,94; IC95% 1,01–3,72) e consumir carne diariamente (OR 7,43; IC95% 1,53–36,11). Por outro lado, consumir açúcar (OR 0,34; IC5% 0,12–0,93) ou azeite de oliva (OR 0,33 IC95% 0,13–0,81) uma ou duas vezes por semana e comer vegetais diariamente (OR 0,39; IC95% 0,15–0,99) foram considerados fatores associados negativamente. Em adultos, a exposição a fungos no domicílio (OR 5,25; IC95% 1,01–27,22) e o consumo de carne uma ou duas vezes por semana (OR 46,45; IC95 2,12–1020,71) foram fatores associados ao diagnóstico médico de rinite alérgica, enquanto a baixa escolaridade (OR 0,25; IC95% 0,07–0,92) se mostrou como fator associado negativamente.

**Conclusões:**

A prevalência de rinite alérgica em adolescentes é alta, assim como seu diagnóstico médico em adultos residentes em Uruguaiana. Fatores ambientais, especialmente hábitos alimentares, foram associados aos achados em ambos os grupos.

## INTRODUCTION

Allergic diseases are common at different stages of life, resulting in high costs of healthcare, school absenteeism, and decreased productivity, thus affecting the quality of life of patients and their caregivers.^1^

Epidemiological studies on the prevalence of allergic diseases have used standardized measuring tools to achieve importance in the recent decades, which brought more knowledge about them. Following the International Study of Asthma and Allergies in Childhood (ISAAC),^2^ the Global Asthma Network (GAN) was established in 2012 with the objectives of understanding the current state of asthma, preventing it, and improving the overall care of asthmatic patients, especially in low- and middle-income countries.^3^

The GAN standard questionnaire was based on the ISAAC phase III^2^ study, which provides similar versions for adolescents (13–14 years old)^4^ and adults (parents and/or guardians).^5^

Allergic rhinitis (AR) is the most common allergic disease in children; however, it is often underestimated because it is a non-life-threatening disorder. In fact, it is a global health problem. Data obtained from the ISAAC phase III study showed that the prevalence of AR in adolescents ranges from 4.5% (Georgia, Europe) to 45.1% (Asunción, Paraguay).^6^ In Latin America, the mean prevalence of AR is 18.5%, ranging from 7.1% (Cuernavaca, Mexico) to 45.1% (Asunción, Paraguay).^7^ In Brazil, there is a variation between 8.9% (São Paulo) and 24.4% (Salvador and Vitória da Conquista).^7^

The ISAAC protocol was retired, showing an increase in the prevalence of allergic rhinitis over 20 years of its application. The GAN protocol opens a new phase of epidemiological assessment of allergic diseases.

This study analyzed the prevalence of the symptoms of rhinitis and the factors associated with its occurrence in adolescents and in their parents/guardians living in the city of Uruguaiana, southern Brazil.

## METHOD

Adolescents and their parents/guardians, living in the city of Uruguaiana (GAN Center No. 504013), participated in a cross-sectional, multicenter, international epidemiological study following the GAN standard protocol.^3^ Between June 2017 and December 2018, 1,200 adolescents from all 17 public schools were invited to participate and answer the GAN phase I^4^ standard questionnaire. The selected public schools were the same that participated in previous study, in addition to private schools that did not agree to participate. Inadequately completed or incomplete questionnaires were excluded, and 1,058 adolescents remained. The adolescents answered the questionnaires in their classrooms under the supervision of a researcher.

The adolescents included took the questionnaires to their parents/guardians (25–75 years old; mean age=42.1 years; standard deviation 8.8 years) to be answered at home and returned within one week. A total of 920 questionnaires were returned, and 896 (97.4%) were answered accordingly.

The study was approved by the Ethics Committee of the Universidade Federal do Pampa (Unipampa), CAAE no. 62658216.2000.5323, and the Informed Consent and Consent Forms were signed by the adolescents and their parents/guardians, respectively.

The GAN standard questionnaires,^4,5^ originally in English, were translated into Portuguese, back translated as recommended, and evaluated for understandability and retention of the contents of the original tool.^8^ Demographic data such as age, sex, date of birth, grade of school, date of interview, current height and weight, and birth weight were included. In addition, nasal problems associated with nasal and eye pruritus affecting daily activities; medical diagnosis of AR; previous and current exposure to paracetamol, antibiotics, and pollution; physical activity; and food consumption were also considered in this study. The questionnaires were coded by the center and the school, and participant number was used to ensure confidentiality and to link the questionnaires answered by the adults (parents/guardians) and their adolescents.^3^

Among the adolescents, an affirmative answer to the question “*In the past 12 months, has this nose problem been accompanied by an itchy nose?*” identified respondents with AR; an affirmative answer to the question “*In the past 12 months, has this nose problem been accompanied by itchy-watery eyes?*” identified respondents with allergic rhinoconjunctivitis; and an affirmative answer to the question “*In the past 12 months, this nose problem interfered moderately/a lot with your daily activities?*” identified respondents with symptoms of severe rhinitis.

In adults, an affirmative answer to the question *“Was your allergic rhinitis confirmed by a doctor?”* identified respondents with AR.

As established by the ISAAC study, the sample size was set between 1,000 and 3,000 participants due to the number of hypotheses investigated, and to get a high response rate, since there are concerns that asthma, rhinitis, or eczema symptoms may result in school absenteeism.^9^

The collected data was entered in an independent double entry into Microsoft Office Excel^®^ database. On comparison of the two entries, no inconsistencies were found. The data was sent to the GAN Center^2^ in Auckland, New Zealand, for initial evaluations, and afterwards, to the GAN Data Center in Spain for further verification.

Categorical variables were presented as frequency distribution and proportions, and continuous variables were presented as mean and standard deviation. Factors associated with AR in the last 12 months were identified by bivariate and multivariate analysis. The introduction of factors in the multivariate analysis considered p-values lower than 0.20 obtained in bivariate analysis, according to the downward binary logistic regression analysis method. P-values lower than 0.05 were considered statistically significant.

## RESULTS

Nasal symptoms were indicated by 61.9% of the adolescents, AR by 28.0%, allergic rhinoconjunctivitis by 21.3%, moderate/severe forms of rhinitis by 7.8%, and 29.7% had a medical diagnosis of rhinitis. In adults, the frequency of medical diagnosis of AR was 31.7%.

The positively associated factors (multivariate analysis) with AR in adolescents are presented in Table 1. The risk factors for AR identified in this study included engaging in exercise once or twice a week; having only one older sibling; daily meat consumption; butter consumption once or twice a week; and cow milk consumption, regardless of the frequency. Negatively associated factors identified were: consuming sugar once or twice a week, daily consumption of legumes (beans, lentils, peas), olive oil consumption once or twice a week, and fast-food consumption every day.

**Table 1.** Factors significantly associated with allergic rhinitis in adolescents, using logistic regression.

	Univariate	Multivariate
		n=335 LR=184.65
	OR (95%CI)	ORa (95%CI)
Physical exercises in a week		
Three or more times	1.54 (1.02–2.33)*	1.43 (0.69–2.97)
Once or twice	1.93 (1.35–2.75)*	2.16 (1.15–4.05)*
Number of old siblings		
One	1.15 (0.79–1.67)	1.94 (1.01–3.72)*
Meat consumption		
Every day	2.51 (1.23–5.12)*	7.43 (1.53–36.11)*
Butter consumption		
Once or twice a week	1.05 (0.67–1.64)	2.05 (1.01–4.16)*
Milk consumption		
Every day	1.26 (0.80–1.96)	2.13 (1.09–4.17)*
Once or twice a week	1.72 (1.06–2.80)*	2.65 (1.23–5.69)*
Sugar consumption		
Once or twice a week	0.58 (0.32–1.05)	0.34 (0.12–0.93)*
Legumes consumption		
Every day	0.73 (0.41–1.30)	0.39 (0.15–0.99)*
Olive oil consumption		
Once or twice a week	0.82 (0.47–1.44)	0.33 (0.13–0.81)*
Fast-food consumption		
Every day	0.64 (0.34–1.20)	0.28 (0.09–0.91)*

n: number of subjects included in analysis; LR: likelihood ratio; OR: odds ratio; ORa: adjusted odds ratio; 95%CI: 95% confidence interval. *significant values (p<0.05).

Table 2 shows the factors significantly associated with the occurrence of allergic rhinoconjunctivitis in adolescents. The positively associated factors identified were: having only one older sibling, using paracetamol at least once a month, eating pasta once or twice a week, and consuming dairy products daily. The negatively associated factors identified were: eating boiled vegetables once or twice a week and bread at least once or twice a week.

**Table 2.** Factors significantly associated with allergic rhinoconjunctivitis in adolescents with rhinitis in the past year, identified using logistic regression.

	Univariate	Multivariate
		n=322 LR=167.15
	OR (95%CI)	ORa (95%CI)
Number of old siblings		
One	1.14 (0.76–1.71)	2.12 (1.09–4.11)*
Use of paracetamol		
Once a month	2.58 (1.60–4.16)*	3.98 (1.85–8.57)*
Boiled vegetables consumption		
Once or twice a week	0.75 (0.49–1.16)	0.48 (0.24–0.97)*
Bread consumption		
Every day	0.70 (0.30–1.64)	0.24 (0.06–0.89)*
Once or twice a week	0.55 (0.22–1.39)	0.16 (0.04–0.66)*
Pasta consumption		
Once or twice a week	2.10 (1.19–3.72)	3.06 (1.22–7.67)*
Dairies consumption		
Every day	1.44 (0.79–2.62)	3.54 (1.16–10.82)*

n: number of subjects included in analysis; LR: likelihood ratio; OR: odds ratio; ORa: adjusted odds ratio; 95%CI: 95% confidence interval. *significant values (p<0.05).

The positively associated factors identified for severe rhinitis were: having only one older sibling and using paracetamol at least once a month (Table 3).

**Table 3.** Significant factors associated with severe rhinitis in adolescents with rhinitis in the past year, identified by logistic regression.

	Univariate OR (95%CI)	Multivariate n=321 LR=101.70
		ORa (95%CI)
Number of old siblings		
One	1.96 (1.09–3.51)*	2.60 (1.05–6.45)*
Use of paracetamol		
Once a month	3.49 (1.51–8.06)*	4.06 (1.38–11.95)*

n: number of subjects included in analysis; LR: likelihood ratio; OR: odds ratio; ORa: adjusted odds ratio; 95%CI: 95% confidence interval. *significant values (p<0.05).

In adults, the positively associated factors for a medical diagnosis of AR were: mold exposure in the household and eating meat once or twice a week. Having low educational level was found as a negatively associated factor (Table 4).

**Table 4.** Factors significantly associated with medical diagnose of allergic rhinitis using logistic regression in adults.

	Univariate	Multivariate
		n=136 LR=58.92
	OR (95%CI)	ORa (95%CI)
Low education		
£8 years in the school	0.47 (0.28–0.77)*	0.25 (0.07–0.92)*
Mold in the household		
Larger than a postcard	2.44 (1.35–4.42)*	5.25 (1.01–27.22)*
Meat consumption		
Once or twice a week	2.25 (0.42–12.03)	46.54 (2.12–1,020.71)*

n: number of subjects included in analysis; LR: likelihood ratio; OR: odds ratio; ORa: adjusted odds ratio; 95%CI: 95% confidence interval. *significant values (p<0.05).

## DISCUSSION

The Uruguaiana GAN center was the first registered Brazilian center to complete a data collection.^5^ The prevalence of AR in adolescents was similar to the medical diagnosis of AR, which was lower than in adults. A phase II ISAAC study conducted 15 years ago in the same city reported a slightly higher prevalence of AR than today.^10^ As for the prevalence of allergic rhinoconjunctivitis, the result corroborated another Brazilian study that used the ISAAC protocol in more than 20,000 adolescents from seven Brazilian locations.^11^

The city of Uruguaiana experienced significant changes in the last 15 years, with the increase of basic sanitation from 16.0% to 81.2%,^12^ which reflected an improvement in the Human Development Index (HDI) from 0.523 to 0.744.^13^

Although it is a small difference, a higher frequency of AR diagnosis was noticed in male adolescents (data not presented); this is different from the data documented in a recent meta-analysis.^14^ The authors documented a predominance in males aged under 11 years and females aged over 12 years, with no difference in the adults. There were more female respondents among the adult/guardian participants; however, it did not allow any inferences regarding the higher prevalence of AR in women since the result could be a response bias.

Prevalence differences favoring post-pubertal women are attributed to the presence of higher levels of endogenous estrogens that determine an increased Th2 response in women. In contrast, testosterone in men acts by suppressing Th2 response.^15^

Epidemiological global studies in adults are rare. The prevalence of AR in US adults ranges between 10 and 30%,^16^ and approximately 40% in Europeans.^17^ The adults evaluated in this study showed a prevalence of AR diagnosis within the mean range.

As for the factors associated with the occurrence of AR symptoms in adolescents, performing minimal exercise was associated with increased AR risk. A study of adolescents using the ISAAC phase III database associated vigorous physical activity (up to three times a week *vs.* none or occasional) with increased frequency of asthma, rhinoconjunctivitis, and eczema symptoms.^18^ Such apparently antagonistic situations could be explained by the fact that shorter exercise times are usually associated with longer sedentary periods, which leads to less physical conditioning and possibly greater exposure to aeroallergens and aggravated respiratory allergic diseases.^19^ In addition, physical activity in a polluted environment may justify its association with respiratory health problems, especially during the practice.^20^ Therefore, many authors recommend not to do vigorous physical activities on high-air-pollution days, especially outdoors. Similarly, little physical activity was also positively associated with increase of allergic rhinoconjunctivitis.

In adolescents, having only one older sibling was positively associated with increase of AR, as proposed by Strachan^21^ and corroborated by other authors,^22,23^ and with allergic rhinoconjunctivitis and severe forms of rhinitis. In summary, frequent contact with viral infections would stimulate the development of standard Th1 immune response.

Although the use of paracetamol has been related to the development of allergic diseases such as asthma and AR,^24,25^ it was not observed in the adolescents evaluated in this study. However, the use of paracetamol at least once a month was identified as a positively associated factor for the occurrence of allergic rhinoconjunctivitis and severe forms of rhinitis.

In adults, mold exposure in the household was identified as a positively associated factor for a medical diagnosis of AR. Household humidity is believed to aggravate AR symptoms and increase susceptibility to common colds and possibly other respiratory infections in young adults.^26^ These results were also observed in a multicenter study conducted in eight Chinese cities, especially among adults living in buildings constructed after 2005.^27^

The relationship between diet and occurrence of allergic diseases, asthma, and AR has been extensively studied. This study shows that diet was one of the main factors associated with AR occurrence. The data show that adolescents who consume meat daily, butter, once or twice a week, and milk, regardless of frequency, and adults who consume meat once or twice a week, were positively associated with increase of allergic disease.

Some studies showed a positive relationship between trans-fat intake, AR, and asthma.^28–30^ Analysis of the ISAAC phase III database found that a Western pattern diet, with high margarine, trans fatty acids, animal fats, and fast-food intakes was a risk factor for AR; while a Mediterranean diet, based on vegetables, cereal, fruit, seafood, peanuts, and olive oil was confirmed as a protective factor.^29^ The results presented in this study found the Western pattern diet as negatively associated, contrasting with the results reported by other authors.^28–31^

Fast-food daily intake was a negatively associated factor, contrasting with the results of ISAAC phase III. Another point to be considered is the difficulty with the use of a food recall survey to assess the consumption of certain foods, especially the processed ones, in terms of presentation diversity, types of preparation, and preservation.^29^

Having a higher educational level could be related to a better socioeconomic level, better knowledge and understanding of the symptoms and the disease, better understanding of the questions and answers, and better opportunities for diagnosis and medical treatment.^32^ Among parents/guardians, the lower educational level was found to be a negatively associated factor for AR occurrence.

One of the limitations of this study is the fact that the information was obtained through a written, self-administered questionnaire focused on symptoms and self-reported AR diagnosis, which was subject to information bias. Verification by physical examination and/or specific laboratory evaluation could reduce such uncertainties. In addition, because it is a cross-sectional study, it is not possible to establish the cause-and-effect relationship with certainty.

This is a local study that represents adolescents only from public schools of a single municipality and therefore may not be extrapolated to general population. However, obtaining basic epidemiological data on allergic diseases in adolescents and adults will enable the development of a global map of these diseases that will improve public health planning and provide better management strategies.

In conclusion, the prevalence of AR in adolescents is high, as well as its medical diagnosis in adults living in Uruguaina. Environmental factors, especially food habits, were associated with findings in both groups.
